# Depicting Biomarkers for HER2-Inhibitor Resistance: Implication for Therapy in HER2-Positive Breast Cancer

**DOI:** 10.3390/cancers16152635

**Published:** 2024-07-24

**Authors:** Alvan Cai, Yuan Chen, Lily S. Wang, John K. Cusick, Yihui Shi

**Affiliations:** 1 College of Medicine, California Northstate University, Elk Grove, CA 95757, USA; alvan.cai9489@cnsu.edu (A.C.); john.cusick@cnsu.edu (J.K.C.); 2 Section Pathology of the Institute of Forensic Medicine, Jena University Hospital, Friedrich Schiller University Jena, Am Klinikum 1, 07747 Jena, Germany; yuan.chen@med.uni-jena.de; 3 University of California, Berkeley, CA 94720, USA; lwang26@berkeley.edu; 4 California Pacific Medical Center Research Institute, Sutter Bay Hospitals, San Francisco, CA 94107, USA

**Keywords:** HER2, breast cancer, anti-HER2-therapy, anti-HER2 therapy resistance, biomarker, combination therapy

## Abstract

**Simple Summary:**

The development of HER2-inhibitors for the treatment of HER2-positive breast cancer represented a breakthrough in targeted tumor therapy. However, as for other targeted therapeutics, drug resistance remains a serious challenge to the treatment of HER2+ BC. Recent research has identified critical biomarkers for HER2-inhibitor resistance and explored more effective treatment regimens in HER2+ breast cancer to overcome drug resistance; however, research on several potential biomarkers and promising alternative therapies remains incomplete.

**Abstract:**

HER2 (human epidermal growth factor receptor 2) is highly expressed in a variety of cancers, including breast, lung, gastric, and pancreatic cancers. Its amplification is linked to poor clinical outcomes. At the genetic level, HER2 is encoded by the ERBB2 gene (v-erb-b2 avian erythroblastic leukemia viral oncogene homolog 2), which is frequently mutated or amplified in cancers, thus spurring extensive research into HER2 modulation and inhibition as viable anti-cancer strategies. An impressive body of FDA-approved drugs, including anti-HER2 monoclonal antibodies (mAbs), antibody–drug conjugates (ADCs), and HER2-tyrosine kinase inhibitors (TKIs), have demonstrated success in enhancing overall survival (OS) and disease progression-free survival (PFS). Yet, drug resistance remains a persistent challenge and raises the risks of metastatic potential and tumor relapse. Research into alternative therapeutic options for HER2+ breast cancer therefore proves critical for adapting to this ever-evolving landscape. This review highlights current HER2-targeted therapies, discusses predictive biomarkers for drug resistance, and introduces promising emergent therapies—especially combination therapies—that are aimed at overcoming drug resistance in the context of HER2+ breast cancer.

## 1. Introduction

Female breast cancer (BC) incidence rates have gradually increased at about 0.5% annually since the mid-2000s [1]. Worldwide, BC stands out as one of the most prevalent cancers, accounting for 31% of female cancers [2]. Of note, one in ten women will be diagnosed with BC in their lifetime [2,3]. It was estimated that the United States would have 297,790 new cases in 2023 [2]. In developed countries, the risk factors for BC include obesity, contraceptive use, late menopause, and late childbirth, all contributing to estrogen genotoxicity. In developing countries, BC is a product of insufficient disease awareness, a lack of screening programs, and limited access to healthcare. While early mammogram screenings, surgical advances, and novel drug discoveries have collectively led to a 43% decrease in BC mortality since its peak in 1989, the five-year survival rate of patients with metastatic BC on adjuvant chemotherapy remains troublingly low at less than 30% [2,4].

Breast cancers exhibit significant diversity and are therefore characterized via histopathology, grade, stage, and immunophenotype [5]. Histopathological classification based on tissue derivation and histological appearance separates cancers into ductal and lobular breast carcinoma. The majority of newly diagnosed cases fall under invasive ductal carcinoma (IDC), with the remaining cases categorized as invasive lobular carcinoma (ILC) [6]. IDC can be further classified as “no specific type” and “specific type”, which includes medullary, metaplastic, apocrine, mucinous, cribriform, tubular, neuroendocrine, classic lobular, and pleomorphic lobular carcinoma [7]. BC staging involves analyzing the progression of local to metastatic cancer at a cellular and organ systems level, while tumor grading reflects tumor cell differentiation. Receptor status is a key determinant, categorizing breast cancers based on the predominant receptor expressed on the surface of the cancer cells. Hormone receptor-positive (HR+) cancers include estrogen receptor (ER+) and progesterone receptor (PR+) cancers, and can be subdivided into luminal A and luminal B cancers. HER2+ cancers overexpress HER2 (human epidermal growth factor receptor 2). Basal-like cancers, or triple-negative breast cancers (TNBC), lack the expression of all three receptors (ER-/PR-/HER2-).

HER2 is a 185kDa transmembrane receptor tyrosine kinase encoded by the ERBB2 gene found on chromosome 17q12. It is part of the epidermal growth factor receptor (EGFR) family, which also comprises EGFR (HER1), HER3, and HER4. Normally, although HER2 itself does not activate from a dedicated ligand interaction, HER2 activation can result from multiple other modalities: homodimerization (HER2/HER2) and heterodimerization (EGFR/HER2, HER2/HER3, and HER2/HER4). HER2 downstream pathways that have interested researchers are the PI3K/Akt/mTOR and NF-kB pathways, both of which induce gene expression for cell proliferation and survival. In the setting of BC cancer, the amplification of HER2 on the surface of BC cells directly enhances the summative effect of HER2 homo/heterodimerization and contributes to sustained pathologic tumor proliferation and survival signals [8].

HER2 overexpression is found in 15–20% of advanced BCs and is related to an aggressive phenotype and unfavorable clinical outcome [9]. Clinically, tumors with a strong staining of HER2 in immunohistochemistry (IHC) staining (more than 10% of tumor cells positive for HER2) or gene amplification detected by fluorescence in situ hybridization (FISH) are defined as HER2 positive, following the guidelines set by the American Society of Clinical Oncology-College of American Pathologists (ASCO-CAP) [10].

HER2+ BCs contain between 25 and 50 copies of the ERBB2 gene which results in a 40–100 fold increase in HER2 receptors (2 million receptors on an HER2+ BC cell). Prior studies analyzing the mechanisms behind HER2 amplification were inconclusive in distinguishing whether amplification resulted from increased double minutes (DMs) or homogenously staining regions (HSRs). Most breast cancers are caused by one method of amplification, but due to the presence of HER2 amplifications characterized by both methods, it was believed that HER2 amplification could be a result of a separate amplification method [11]. Recent studies have found HER2 amplifications to be characterized by palindromic repeats generated from Breakage–Fusion–Bridge cycles [12].

HER2-targeted therapies encompass monoclonal antibodies (mAbs), tyrosine kinase inhibitors (TKIs), and antibody–drug conjugates (ADCs) [8]. Trastuzumab, the first FDA-approved anti-HER2 mAb (approved 1998), greatly advanced the prognostic landscape for HER2+ BC [13]. The success of trastuzumab facilitated the development of other HER2-targeted therapies [9]. As of now, the FDA has approved eight HER2-targeted agents, including three anti-HER2 mAbs (trastuzumab, pertuzumab, and margetuximab), two ADCs (T-DM1 and T-DXd), and three TKIs (lapatinib, neratinib, and tucatinib) [9]. The history and development of HER2 inhibitors were addressed in detail in a recent review article [14].

Like other targeted therapeutic strategies, both innate and acquired drug resistance pose significant challenges for HER2-targted therapy. Understanding and identifying the mechanisms and biomarkers involved in drug resistance may play a key role in stratifying patients for personalized therapy. This review consolidates the existing literature to provide a comprehensive overview of the standard of care for HER2+ BC, mechanisms and biomarkers of drug resistance, and approaches to combat drug-resistant HER2+ BC. 

## 2. Standard of Care for HER2+ BC

### 2.1. Monoclonal Antibodies (mAbs)

#### 2.1.1. Trastuzumab

Over the past two decades, the development of targeted therapies for HER2+ BC has flourished, significantly advancing the prognostic landscape for HER2+ BC patients. In 1998, trastuzumab (Herceptin^®^) was approved by the FDA as the first targeted therapeutic drug for metastatic HER2+ BC, and the first humanized monoclonal antibody targeted against the HER2 protein [15]. Although the scope of molecular therapies has expanded tremendously since then, trastuzumab remains the gold standard for the treatment of advanced-stage HER2+ BC. Numerous randomized trials have favored trastuzumab-containing regimens for better overall survival (OS) and disease-free survival (DFS), especially when trastuzumab is used as an adjuvant therapy [16,17,18,19,20,21]. Thus, trastuzumab’s role in first-in-line treatment (although often in combination with pertuzumab, another mAb, and chemotherapy) for both adjuvant and metastatic HER2+ BC is well deserved.

While trastuzumab has also been proven predominantly safe, trastuzumab-induced cardiotoxicity, such as dilated cardiomyopathy, remains the most frequent adverse event, with a documented decrease in LVEF ≥10% in 22% of patients. This risk has prompted many trastuzumab studies to list normal or above-normal baseline cardiac function as a prerequisite for study entry [22]. Patients with preexisting cardiac issues are thus precluded from treatment with trastuzumab. The proactive management of cardiac function remains critical during treatment [23]. Fortunately, with regular cardiac monitoring, the incidence of heart failure has significantly decreased [24,25,26]. Nevertheless, trastuzumab carries an FDA-boxed warning of infusion reaction-induced adverse effects such as angioedema, anaphylaxis, interstitial pneumonitis, and acute respiratory distress syndrome [27]. Other side effects related to the monotherapy of trastuzumab include gastrointestinal disorders, skin disorders, headache, chills, and fatigue [28].

**Figure 1:**
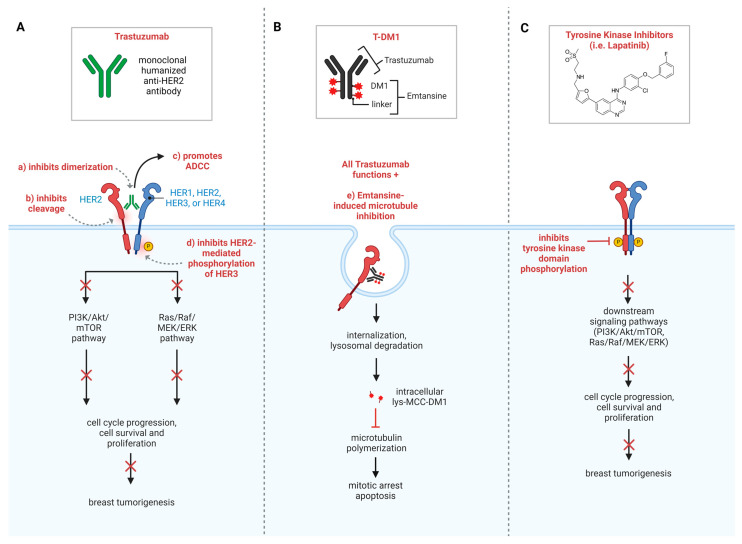
Current standard of care for HER2+ breast cancer. (**A**) Trastuzumab is a multi-functional treatment option that inhibits HER2 dimerization, prohibits phosphorylation, and promotes antibody-dependent cellular cytotoxicity (ADCC). (**B**) Antibody–drug conjugates (ADCs) operate with similar binding functionality as trastuzumab with the added effect of targeted cytotoxic payloads. (**C**) Tyrosine kinase inhibitors (TKIs) inhibit phosphorylation of tyrosine kinase residues, preventing activation of downstream signaling pathways.

Despite clear clinical benefits, the mechanisms of action of trastuzumab have spurred significant debate throughout decades of research. It is commonly accepted that trastuzumab’s anti-tumor effects primarily function through its binding to the extracellular domain (ECD) IV of HER2, thus blocking ligand-independent HER2 dimerization and inducing antibody-dependent cell-mediated cytotoxicity (ADCC) [29,30] (Figure 1A) Other proposed mechanisms of action comprise the suppression of downstream oncogenic pathways, activation of the immune system, induction of cell cycle arrest and apoptosis, and inhibition of DNA repair [31,32,33].

Trastuzumab has been shown to inhibit the PI3K/AKT signaling pathway by suppressing AKT phosphorylation via the activation of PTEN phosphatase by Src inhibition and via the inhibition of the HER2-mediated phosphorylation of HER3, inducing cell cycle arrest [34,35,36,37]. Additionally, a growing body of evidence from preclinical and clinical studies indicates that the immune system contributes substantially to the HER2-targeted therapy [38]. Complex interactions have been identified among different branches of the immune system, HER2+ BC, and targeted treatments [39]. Finally, the HER2 pathway plays a role in repairing DNA damage induced by chemotherapeutic agents. Trastuzumab delays the repair of the interstrand crosslinks produced by cisplatin in the breast cancer cell lines MCF-7, SK-BR-3, and MDA-MB-453 [40]. Extensive research has divulged numerous auxiliary functions of trastuzumab, including the inhibition of tumor-favored angiogenesis [41], activation of antibody-dependent cellular phagocytosis by macrophages [42], and activation of the classical complement pathway [43], giving rise to complement-dependent cytotoxicity and phagocytosis.

#### 2.1.2. Pertuzumab

The discovery of trastuzumab heralded the current era of precision medicine in HER2+ BC, paving the way for new targeted therapies modeled for HER2+ BC. In 2012, pertuzumab (Perjeta^®^) was FDA approved for use with trastuzumab and docetaxel, a microtubule depolymerization inhibitor, for first-line therapy for HER2+ metastatic BC (mBC) [44]. Pertuzumab is a recombinant humanized mAb that binds to the extracellular domain II (ECD2) of HER2, thus targeting a different epitope from that of trastuzumab [45,46] (Table 1). Pertuzumab with trastuzumab and chemotherapy was further approved in the neoadjuvant setting in 2013, and in the adjuvant setting in 2017 for early-stage HER2+ BC with a high risk of recurrence [47,48]. The clinical studies involved with each approval (CLEOPATRA, NeoSphere and TRYPHAENA, and APHINITY, respectively) evidenced significant advances in progression-free survival (PFS) and OS, and also developed a good safety profile for pertuzumab similar to that of trastuzumab [46].

The synergistic efficacy of pertuzumab with trastuzumab is substantiated by its distinct binding epitope (ECD2, as opposed to ECD4, the binding domain of trastuzumab). Additionally, pertuzumab was thought to induce a more complete blockage of HER2 activation via the inhibition of HER2 heterodimerization with HER1, HER3, and HER4, complementing trastuzumab’s inhibition of HER2 homodimerization to block downstream oncogenic signaling pathways [46,49]. Recently, it has been demonstrated that pertuzumab exhibits a slightly higher binding affinity to HER2 than trastuzumab, as estimated by a PRODIGY model [50]. However, further research is needed to elucidate the additional mechanisms of action pertuzumab may possess once it has bound to HER2.

#### 2.1.3. Margetuximab

More recently, margetuximab (MARGENZA^®^, margetuximab-cmkb), a chimeric monoclonal antibody, was FDA approved in 2020 for pre-treated HER2+ mBC patients [51] (Table 1). Margetuximab shares HER2 specificity with trastuzumab, binding to ECD4, but distinguishes itself in that the Fc portion of the antibody was engineered to enhance the ADCC of margetuximab-bound BC cells by the innate immune system relative to trastuzumab [52]. In the phase 3 SOPHIA trial associated with FDA approval, the combination of margetuximab with chemotherapy was shown to induce a moderate benefit in PFS vs. trastuzumab combined with chemotherapy; however, there was no significant improvement in OS [53,54]. Ongoing studies are currently exploring potential benefits from margetuximab alone or in combination with PD-1/PD-L1 inhibitors in HER2+ BC and also in other solid tumors [55].

### 2.2. Antibody–Drug Conjugates (ADCs)

Antibody–drug conjugates (ADCs) represent a pioneering class of drugs in cancer treatment. These specialized compounds are engineered to harness the specificity of mAbs by combining them with potent cytotoxic agents, thus offering a targeted and more precise approach [56]. More specifically, the antibody component of ADCs selectively binds to a biomarker/protein (such as HER2) on the surface of the cancer cells, and the covalently bound cytotoxic agent is subsequently released, leading to cell death [57] (Figure 1B, Table 1). Thus, ADCs enhance therapeutic efficacy by minimizing off-target effects in cancer treatments.

#### 2.2.1. T-DM1 (Trastuzumab Emtansine)

The first ADC for HER2+ BC, trastuzumab emtansine (T-DM1, or Kadcyla^®^), was FDA approved in 2013 for HER2+ mBC patients who previously received trastuzumab and taxane [58] (Table 1). T-DM1 consists of a trastuzumab mAb conjugated with 3–4 emtansine (DM1) moieties via non-cleavable thioether linkers. T-DM1 functions through multiple mechanisms of action, including the selective delivery of DM1 to the HER2+ tumor cell, trastuzumab-mediated suppression of the HER2 signaling pathway, inhibition of HER2 extracellular domain shedding, and induction of antibody-dependent cell-mediated cytotoxicity (ADCC) [59]. In the EMILIA trial, upon which the FDA approval was based, T-DM1 demonstrated significant clinical efficacy (higher PFS and OS) and lessened toxicity in comparison to lapatinib plus capecitabine, a standard of care, in HER2+ BC patients exhibiting disease progression with trastuzumab and a taxane [60]. In the KATHERINE trial, T-DM1 demonstrated significant clinical efficacy (in terms of recurrence) in comparison to trastuzumab alone, in the early-stage disease setting after neoadjuvant treatment [61]. These results were confirmed in the TH3RESA trial in comparison to treatment of physician’s choice [62]. However, both the 2016 MARIANNE study and the 2017 KRISTINE trial showed non-superior efficacy for T-DM1 with pertuzumab as a first-line treatment and in the neoadjuvant setting, respectively, although T-DM1 was better tolerated than the standard of care [63].

Due to structural similarities to trastuzumab, T-DM1 treatment exhibits similar adverse effects as those found with trastuzumab treatment, most notably cardiotoxicity and decreased LVEF. In addition, approximately 30% of patients who underwent T-DM1 treatment experienced thrombocytopenia treatable via T-DM1 dose reduction in two separate studies [64,65].

Overall, T-DM1 represents a novel therapeutic strategy for HER2+ BC via its selectivity of tumor cells for a potent cytotoxic payload.

#### 2.2.2. T-DXd (Trastuzumab Deruxtecan)

While the development of targeted therapies for HER2+ BC has thrived in the past few decades, the treatment options for patients with HER2-low BC remain stagnant. In most of the clinical trials, HER2-low status is defined as tumors with an HER2 IHC score of 1+ or 2+ without *ERBB2* amplification [66]. More than 50% of BCs are currently defined as HER2-low BC [67]. The development of trastuzumab deruxtecan (T-DXd, formerly DS-8201a, or Enhertu^®^) thus represented a crucial breakthrough in advancing the bleak prognostic landscape for HER2-low cancers. In 2022, the FDA approved Enhertu for adult patients with HER2-low mBC who have received prior chemotherapy in the metastatic setting or developed disease recurrence during or within six months of completing adjuvant chemotherapy. The primary risks associated with T-DXd include interstitial lung disease and pneumonia [68].

T-DXd consists of a trastuzumab mAb conjugated to the novel exatecan-derivative topoisomerase I inhibitor DXd via a peptide linker cleavable by lysosomal enzymes, which are highly expressed in tumor cells and their microenvironment [69]. Its cleavable link differentiates T-DXd from T-DM1 in that it allows T-DXd to release its cytotoxic payload into neighboring tumor cells lacking HER2 expression via a bystander effect. Additionally, T-DXd incorporates a significantly higher drug-to-antibody ratio (DAR) in comparison to T-DM1—an increase from 1:3.5 to 1:8 [70]. T-DXd’s cleavable linker and high DAR can target a wide range of HER2-expressing tumors, additionally allowing it to overcome resistance to T-DM1 [59]. Most critically, its discovery unlocks access for HER2-low patients to the much-needed advantages of novel targeted therapies.

#### 2.2.3. SYD985 (Trastuzumab Duocarmazine)

Trastuzumab duocarmazine (SYD985) was developed almost concurrently alongside T-DXd. Like T-DXd, it is referred to as a “second-generation ADC”. SYD985 consists of trastuzumab conjugated to a duocarmycin derivative, a potent DNA-alkylating agent that intercepts DNA replication to induce cell death, through a novel seco-DUBA linker [71]. SYD985 is highly effective in treating tumors through the bystander killing effect and has also been shown to overcome resistance to T-DM1 [72]. In the phase 3 TULIP trial, the SYD985-treated group had longer PFS than the TPC (treatment of physician’s choice) group (7.0 months vs. 4.9 months) with a statistically significant HR of 0.64, but no benefit regarding OS in HER2+ mBC [73]. SYD985 exhibited a manageable safety profile yet is currently awaiting approval after receiving a complete response letter from the FDA in 2023.

### 2.3. Tyrosine Kinase Inhibitors (TKIs)

In recent years, the development of tyrosine kinase inhibitors (TKIs), including lapatinib, eratinib, tucatinib, and pyrotinib, has played a pivotal role in the treatment of HER2+ BC. These small molecules target the intracellular catalytic kinase domain of HER2 and inactivate downstream oncogenic pathways, such as the PI3k/AKT and mitogen-activating protein kinases (MAPK) pathways, ultimately leading to tumor cell death (Figure 1C, Table 1). Of these, three TKIs—lapatinib, neratinib, and tucatinib—have gained FDA approval for use in either the metastatic or adjuvant setting [8]. Lapatinib is a reversible inhibitor of HER1 and HER2, while neratinib and pyrotinib are irreversible pan-HER TKIs, targeting HER1, HER2, and HER4 [74]. Unlike the other three TKIs, tucatinib functions specifically against HER2. Lapatinib and tucatinib have been approved for treatment of HER2+ mBC, while neratinib has been approved by the FDA for treatment of early-stage HER2+ BC.

TKIs prove most useful in the context of brain metastatic BC. The recurrence of HER2+ BC frequently involves the central nervous system (CNS), leading to brain metastases and unfavorable clinical outcomes in up to 50% of patients [75]. Because HER2 mAbs are large tetrameric proteins, they exhibit a limited ability to cross the blood–brain barrier [76,77]. TKIs, with their smaller size and comparable anti-HER2 activity, exhibit enhanced CNS penetrance.

In the phase 2 LANDSCAPE trial, combination therapy with lapatinib and capecitabine demonstrated an intracranial response in nearly 66% of patients with untreated breast cancer brain metastases [78]. In the NEfERT-T randomized clinical trial (neratinib plus paclitaxel vs. trastuzumab and paclitaxel), 8.3% of patients in the neratinib arm had symptomatic/progressive CNS disease, in contrast to 17.3% of the trastuzumab arm (*p* = 0.002) [79]. Tucatinib, which showed CNS penetration in intracranial xenograft models [80], is currently being investigated in clinical trials. Specifically, the TBCRC049 study, an investigator-initiated phase 2 single-arm study clinical trial enrolling to evaluate the safety and efficacy of tucatinib, trastuzumab, and capecitabine in HER2+ BC with newly diagnosed lymph node metastasis, demonstrated that tucatinib distributed into the cerebrospinal fluid [81]. Other ongoing clinical trials are investigating tucatinib for the treatment of HER2+ mBC with the aim of postponing CNS progression (NCT05132582) and for the management of residual disease compared with T-DM1 (NCT04457596) [8]. Pyrotinib in combination with capecitabine has been approved in China for the treatment of HER2+ mBC previously treated with trastuzumab and taxane [82], and a PANDORA phase 2 trial demonstrated that pyrotinib plus docetaxel had an acceptable safety profile and promising anti-tumor effect as a first-line treatment option for patients with HER2+ mBC [83]. In a phase 3 clinical trial, combination therapy with pyrotinib, trastuzumab, and docetaxel showed significantly improved PFS with manageable toxicity compared to placebo, trastuzumab, and docetaxel in untreated HER2+ mBC, indicating the utility of the dual anti-HER2 regimen as an alternative first-line treatment option [84]. 

The adverse effects of TKIs tend to be multisystem due to the nonspecific targeting of tyrosine kinase receptors. These effects include headaches, skin rashes, and GI symptoms such as grade 3 diarrhea. Lapatinib and neratinib have been associated with cardiotoxic effects such as congestive heart failure and reduced LVEF, both adverse effects found in trastuzumab treatment as well. Pulmonary hypertension as a result of left ventricular dysfunction has been shown as well [85]. Tucatinib adverse effects include elevated AST, ALT, and creatinine levels, palmar–plantar erythrodysesthesia syndrome, and diarrhea [86].

Other irreversible pan-HER inhibitors such as canertinib, proziotinib, epertinib, varlitinib, and TAS0728 are in preclinical or clinical studies [87,88,89]. These advancements demonstrate the evolving landscape of TKIs in the treatment of HER2+ BC, offering diversified therapeutic options with encouraging clinical outcomes.

### 2.4. Summary of Current HER2+ Therapies

As of now, the FDA has approved eight HER2-targeted agents, including three anti-HER2 mAbs (trastuzumab, pertuzumab, and margetuximab), two ADCs (T-DM1 and T-DXd), and three TKIs (lapatinib, neratinib, and tucatinib). Trastuzumab, the revolutionary HER2-inhibitor, remains the gold standard for first-line therapy. T-DXd has also performed admirably, earning its place as the preferred second-line therapy, as well as securing a new role as an effective HER2-low therapy. Tucatinib represents the best of the TKIs so far, outperforming lapatinib and neratinib in both efficacy and safety, and exhibiting significant CNS penetrance in comparison to trastuzumab and other mAbs. Meanwhile, pertuzumab’s high synergy with trastuzumab greatly improves outcomes in all settings. Neratinib and T-DM1 remain the only approved TKI and ADC, respectively, for the adjuvant setting in early BC. Lapatinib has generally been overshadowed by newer TKIs, and margetuximab, while mechanistically promising, has generally failed to surpass trastuzumab’s efficacy. 

These comparisons are detailed in Table 1. Additionally, Table 2 details the mechanisms of action for each drug, as well as the mechanisms for/against resistance.

**Table 1:** FDA-approved HER2 inhibitors in the treatment of HER2+ BC.

Drug (Brand Name)	Type	Route	Approval Year	Treatment Setting	Practical Implications	
					Clinical Benefits	Clinical Shortcomings *
trastuzumab (Herceptin)	mAb	IV	1998	metastatic BC	fairly well tolerated gold standard first-line therapy: although HER2+ treatments have diversified greatly, trastuzumab remains integral to therapy, especially in combination with new generations of drugs	cardiotoxicity is the main ADR of interest, but is manageable with early detection and monitoring [24,25,26] susceptible to resistance limited CNS penetrance [90]
			2006	adjuvant setting		
lapatinib (Tykerb)	TKI	oral	2007	metastatic BC	penetrates into CNS [76,77] in combination with trastuzumab, significantly increases pCR vs. trastuzumab alone [91]	has generally been overshadowed by newer TKIs ADRs include significant diarrhea and rash [85]
pertuzumab (Perjeta)	mAb	IV	2012	metastatic BC	highly synergistic with trastuzumab; significantly improves PFS and OS vs. trastuzumab alone with little difference in serious AEs [92,93]	may increase cardiac burden compared with trastuzumab [94] like trastuzumab, similarly susceptible to resistance and has limited CNS penetrance [90]
			2013	neoadjuvant setting		
			2017	adjuvant setting for early BC		
T-DM1 (Kadcyla)	ADC	IV	2013	metastatic BC	overcomes resistance to trastuzumab [95] and lapatinib [96] greatly outperforms trastuzumab in adjuvant setting (50% decrease in recurrence/death) [61]	may increase AE incidence in comparison to trastuzumab; severe thrombocytopenia is particularly noteworthy [65]
			2019	adjuvant setting for early BC		
neratinib (Nerlynx)	TKI	oral	2017	adjuvant setting for early BC	only TKI for early BC outperforms lapatinib (with CHT), with improved PFS and time to CNS intervention [97] greater potency vs. other TKIs in biochemical assays due to irreversible binding [74]	more severe diarrhea than other TKIs [97]
			2020	metastatic BC		
T-DXd (Enhertu)	ADC	IV	2019	metastatic BC	preferred second-line treatment in metastatic setting; outperforms T-DM1 [59,98] first targeted drug for HER2-low BC [98]	ILD (seen in 10%–15% patients) [68]
tucatinib (Tukysa)	TKI	oral	2020	metastatic BC	strongest activity for CNS metastases [99,100] in comparison with other TKIs, decreased GI and skin toxicities due to higher specificity for HER2 [101]	ADRs include diarrhea and rash [86]
margetuximab (Margenz)	mAb	IV	2020	metastatic BC	improved PFS vs. trastuzumab with chemotherapy [53,54] increased ADCC activity vs. trastuzumab [52]	no significant difference in OS vs. trastuzumab [54] similar cardiotoxicity issues as with trastuzumab [54] and pertuzumab

ADRs: adverse drug reactions; mAB: monoclonal antibody; TKI: tyrosine kinase inhibitors; BC: breast cancer; IV: intravenous; pCR: pathologic complete response; PFS: progression-free survival; OS: overall survival; CNS: central nervous system; CHT: chemotherapy; GI: gastrointestinal; ILD: interstitial lung disease; * ADRs shared among treatments (fever, nausea/vomiting, fatigue, diarrhea) are largely omitted. Drug-specific ADRs and ADRs of greater interest are emphasized instead.

## 3. Biomarkers for HER2 Therapy Resistance

Despite the reported successes of current HER2+ breast cancer (BC) therapies, drug resistance remains a significant challenge, often rendering treatment options ineffective. Drug resistance manifests in two forms: primary resistance, or *de novo* resistance where patients fail to respond to initial therapy; and secondary resistance, or acquired resistance, which arises in patients over the course of treatment due to tumor mutations or alterations in the tumor microenvironment (TME). Within the HER2+ BC population, primary resistance to trastuzumab therapy, the first-line defense against HER2+ BC, arises in 35% of cases [102]. Moreover, among those who do initially respond to trastuzumab therapy, approximately 70% of patients develop mBC within a year [103]. These statistics emphasize the need for new therapeutic approaches to combat drug-resistant breast cancers.

**Figure 2:**
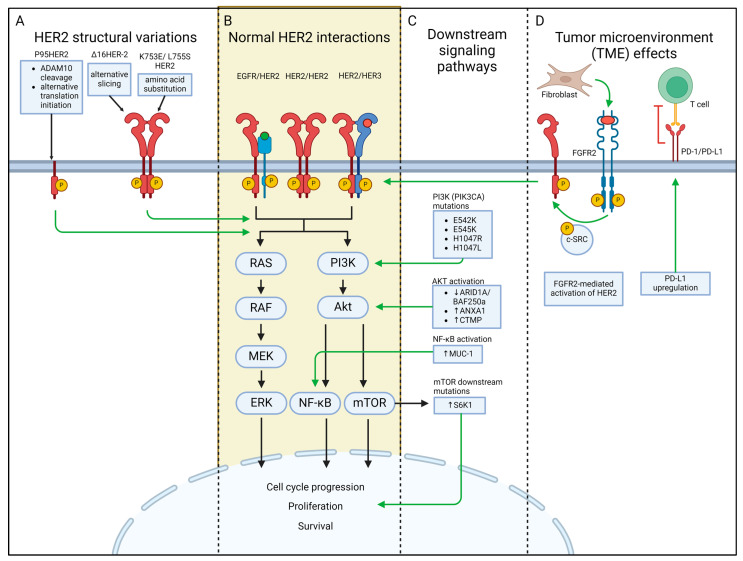
Classification of HER2+ breast cancer therapy-resistant biomarkers. Biomarkers for resistance are classified at the receptor level, within downstream signaling pathways, and inside the tumor microenvironment. (**A**) Receptor-level biomarkers are structural variations that result from cleavage, mutations, or splicing differences, leading to increased receptor activity and subsequent tumorigenesis. (**B**,**C**) Mutations that modify normal PI3K/Akt/mTOR and RAS/RAF/MEK/ERK pathways predispose HER2+ cells to abnormal cell progression, proliferation, and survival. (**D**) Tumor microenvironments contribute to the development and maintenance of tumors via mechanisms such as fibroblast-mediated activation of HER2 and increased PD-L1 expression for T cell immunosuppression.

The mechanisms for HER2-targeted therapy resistance are complex. It is now widely recognized that a variety of factors contribute to anti-HER2 therapy resistance, including HER2 family alterations, the loss or masking of the HER2 epitope, the activation of downstream oncogenic signaling pathways, HER2 heterogeneity, epigenetic dysregulation, the remodeling of the TME, and metabolic reprogramming [8,104] (Figure 2). Current research efforts are striving to identify HER2+ BC biomarkers that can predict treatment response to trastuzumab and other anti-HER2 therapies. Here, we explore such biomarkers both at the receptor level and at the downstream signaling pathway level.

### 3.1. HER2 Structural Variations

Mutations, alternate splicing, and structural modifications at the receptor level have been widely studied for drug resistance in HER2+ BC (Figure 2A). Due to the extensive use of trastuzumab in the standard treatment for HER2+ BC and gastric HER2+ adenocarcinomas [30], resistance to trastuzumab was first attributed to HER2 differences. The same receptor differences that confer trastuzumab resistance are also frequently implicated in other monoclonal antibodies (mAbs) and their derivatives due to the inability of these drugs to successfully antagonize HER2.

#### 3.1.1. P95HER2

One of the most studied biomarkers for drug-resistant HER2+ BC is p95HER2 (95–100 kDa), a truncated, constitutively active version of HER2 (185 kDa) [105]. These carboxy-terminal fragments (CTFs) are a result of both the metalloprotease-mediated shedding of HER2 and alternative initiation of mRNA translation [106]. P95HER2 is associated with a poor prognosis for HER2+ BC, with a median OS of 22.5 months compared to an OS of 35 months in p95HER2-negative patients [107]. Without extracellular binding domains for trastuzumab and pertuzumab, p95HER2 also confers resistance to several current mAb treatments through the constitutive homodimerization and subsequent phosphorylation of the Akt and MAPK pathways [108,109]. In a study examining the relation between nodal metastasis and p95HER2 expression, it was found that p95HER2 was expressed in 29.1% of patients with 1–3 metastatic nodes and in 36.7% of patients with >4 metastatic nodes [110]. Importantly, a separate study found that only 11.1% of p95HER2-expressing patients responded to trastuzumab treatment compared to a 51.4% response rate of full-length HER2-expressing patients [108].

Due to the prevalence of HER2 shedding, current research is concerned with investigating the mechanisms behind this occurrence. Dolichyl-phosphate N-acetylglucosaminephosphotransferase 1 (DPAGT1) is an endoplasmic reticulum-associated protein critical for the maturation and stability of ADAM10. ADAM10, if protected from endoplasmic reticulum-associated degradation, can promote the shedding of the HER2 ectodomain leading to the formation of p95HER2 and, consequently, trastuzumab resistance [111]. Importantly, the inhibition of DPAGT1 by tunicamycin resensitized HER2+ BC cells to trastuzumab. This result provides a promising insight into the potential mechanism behind HER2 truncation and future studies are needed to further exploit the prevention of HER2 shedding to maintain trastuzumab sensitivity in HER2+ BC [111].

#### 3.1.2. Δ16HER-2

Receptor morphological changes at the level of mRNA splicing can influence HER2+ BC mAb resistance. Δ16HER-2 is the product of an exon 16 deletion in HER2 mRNA, leading to an HER2 extracellular conformation change that favors homodimerization and subsequent activation via disulfide bonding [112]. With a promising potential to act as a biomarker for “stemness” due to its activation of the epithelial–mesenchymal transition (EMT), Wnt, and Notch pathways, Δ16HER-2 is prevalent in 90% of HER2+ mBC [113]. Additionally, the activation-induced coupling of Src kinase to the Δ16HER-2 tyrosine kinase domain (observed in 44% of Δ16HER-2 patients) can be successfully targeted by TKIs such as dasatinib [114] and lapatinib [115]. In vitro studies found that Δ16HER-2 homodimerization also induces resistance to both T-DM1 [116] and trastuzumab, the latter of which can be reversed by emodin, a TKI against HER2 [117]. 

However, a more recent in vivo study has found that Δ16HER-2 does not confer trastuzumab resistance in xenografted mice expressing Δ16HER-2 in the immortalized human breast epithelial cell line MCF10A [118]. Surprisingly, Δ16HER-2 increases the susceptibility of MCF10A cells in mice to trastuzumab. The results of this study were confirmed clinically in HER2+ BC [119] and in HER2+ gastrointestinal cancer patients [120]. Collectively, although Δ16HER-2 is associated with mBC, the effects of Δ16HER-2 on patients with trastuzumab therapy are currently unresolved and warrant further investigation. 

#### 3.1.3. HER2 K753E and L755S

The HER2 K753E mutation has recently been discovered to confer lapatinib resistance to HER2+ BC. Lapatinib is a small TKI for both EGFR and HER2 that prevents EFGR/HER2 downstream signal transduction pathways. Root mean square deviation (RMSD) analysis revealed that lapatinib binds to the HER2 K573E mutation with higher affinity than to wild-type (WT) HER2. Furthermore, lapatinib binds to HER2 K753E in a reverse orientation, essentially conferring lapatinib resistance in cells [121]. Prior studies have shown that the HER2 L755S mutation induces trastuzumab and lapatinib resistance [122,123]. Lapatinib IC50 in HER2 L755S-positive cells was 30-fold higher than the lapatinib IC50 in WT HER2+ BC [123]. By destabilizing and stabilizing the inactive and active forms of HER2, respectively, the HER2 L755S mutation constitutively activates HER2 and renders lapatinib ineffective due to the drug’s normal targeting of the inactive form of HER2. Through in vitro studies [124] and molecular dynamics studies [121], K753E has been theorized to confer lapatinib resistance simply by being in close proximity to L755S. The MEK inhibitor AZD6244 and PI3K inhibitor pictilisib (GDC-0941) synergistically promoted the robust killing of MCF10A cells overexpressing the HER2-L755S mutation [125]. CHMFL-26, which functions as an irreversible kinase inhibitor of several forms of mutated HER2 including L755S and p95HER2, was found to be able to effectively suppress tumor proliferation via the activation of apoptotic pathways both in vitro and in vivo [126].

### 3.2. Downstream Signaling Proteins

Several downstream signaling pathways of HER2 are of interest in biomarker research (Figure 2B). The mutations to these pathways are indicative of increased malignant potential due to unopposed cell cycle continuation. Since monoclonal antibodies (i.e., trastuzumab) and their drug conjugates (i.e., T-DM1) target HER2 extracellular binding sites, mutations that constitutively activate downstream cytoplasmic signaling pathways have the potential to circumvent these receptor-targeted therapies (Figure 2C).

#### 3.2.1. PI3K/Akt/mTOR Pathway

##### PI3KCA H1047R and E545K

One of the common genomic alterations observed in BC is an abnormality in the PI3K/AKT/mTOR signaling pathway. Within the PI3K/Akt/mTOR pathway, the PI3K family is divided into three classes (Class I (Ia, Ib), Class II, Class III), with Class Ia as the mutated form in cancer. The PIK3CA gene is translated into the p110a catalytic subunit of Class Ia PI3K as a 1068 amino acid protein (Vanhaesebroeck et al., 2010). In a study by Martinez-Saez et al., PIK3CA mutations were found in 2261 out of 6338 BC patients (35.7%). PIK3CA “hotspot” mutations include E542K, E545K, H1047R, and H1047L, all of which are gain-of-function mutations, with H1047R being the most prevalent (35%) [127].

In mouse models, H1047R induced multipotency during tumorigenesis in the mammary gland [128]. Mice co-expressing human HER2 and mutant PIK3CA developed tumors with shorter latency times compared with mice expressing either oncogene alone. Additionally, they displayed resistance to trastuzumab and resistance to anti-HER2 combinatorial therapies [129]. In a separate study, mice expressing doxycycline-inducible H1047R PI3K notably increased phosphorylated Akt concentrations and generated FoxO1/FoxO3a, both indicative of PI3K/Akt activation. The tumors in these mice displayed resistance to lapatinib treatment while doxycycline treated, but were susceptible to treatment after the withdrawal of doxycycline, demonstrating that H1047R is a driving force for lapatinib-resistant tumor progression [130]. Similarly, H1047R and E545K mutants were found to be resistant to lapatinib treatment yet were susceptible to dactolisib [131]. E542K and E545K introduce amino acid charge changes that cause the PI3K mutant p85a nSH2 domain to detach from the p110a helical domain, thus separating the regulatory subunit from the catalytic subunit, resulting in constitutively activated PI3K [132,133].

##### S6K1 Overexpression

S6K1, an mTOR downstream target, has recently been shown as a potential biomarker of interest for de novo trastuzumab resistance in HER2+ BC. S6K1 is a ribosomal protein that promotes ribosome function and the translation of many proteins needed for cell growth and proliferation, such as IRS1 (Insulin Receptor Substrate-1) and cyclin D1 [134]. S6K1 expression increased 1.5-fold after trastuzumab treatment in trastuzumab-resistant Δ16HER-2 BC cells, and this result was not replicated under treatment in trastuzumab-sensitive WT HER2 BC cells. Furthermore, this effect was abolished following the treatment of the mTORC1 inhibitor rapamycin, indicating rapamycin might sensitize trastuzumab under the condition of S6K1 overexpression [135]. Several S6K1 inhibitors are currently being developed with promising results in treating S6K1-overexpressing cancers. These drugs include PF-4708671 and FS-115 for the treatment of triple-negative BC, LY2584702 for non-small cell lung cancer, and RAME for cervical cancer [134].

##### ARID1A/BAF250a and ANXA1

The AT-rich interactive domain protein 1A (ARID1A)/BRG1-related factor 250a (BAF250a) is a non-catalytic subunit crucial for the binding of SWItch/Sucrose Non-Fermentable (SWI/SNF) chromatin-remodeling complexes to DNA in nucleosomes. SWI/SNF acts as both a repressor and an activator of gene transcription, and mutations to the complex have been shown in 20–25% of all human cancers [136], with the gene for ARID1A being the most frequently mutated gene in the SWI/SNF complex. For example, it was recently demonstrated that ARID1A mutations confer intrinsic and acquired resistance to cetuximab treatment in colorectal cancer [137]. ARID1A loss of function mutations prevent the proper tumor-suppressing function of the SWI/SNF complex. Also, because ARID1A induces p53/p21 activity to cause cell cycle arrest, studies show that the low expression of ARID1A results in breast cancer progression [138]. Interestingly, by unknown mechanisms, the complete loss of ARID1A was reported to induce annexin-A1 (ANXA1)-mediated activation of the PI3K/Akt/mTOR pathway, resulting in resistance to both trastuzumab and the mTOR inhibitor AZD8055 [139]. Conversely, a separate group subsequently reported a direct correlation between ARID1A expression and ANXA1 expression, and that although ANXA1-positive HER2+ BC tumors exhibited a mortality rate three times higher than that of ANXA1-negative tumors, ANXA1 activity has no prognostic importance in determining trastuzumab resistance [140]. Based on the results, the researchers claimed that the increased expression of ARID1A was a biomarker for poor prognosis in HER2+ BC, a claim supported by some [141] but contested by others [142,143], although it should be noted that the definition of “resistance to trastuzumab” varied slightly between different studies. Clearly, more research is required to resolve the complex interplay between ARID1A, ANXA1, and trastuzumab resistance in HER2+ BC.

##### CTMP Overexpression

The Carboxyl Terminal Modulatory Protein (CTMP) is highly upregulated in BC, is associated with poor prognosis and early recurrence [144], and is associated with increased metastasis in mouse models [145]. Although initially reported to be a negative regulator of Akt phosphorylation [146], CTMP was subsequently shown to bind Akt and promote the phosphorylation and activation of Akt [147,148]. When two HER2+ cell lines, SK-BR-3 (low CTMP) and BT-483 (high CTMP), were treated with trastuzumab, the high-CTMP BT-483 cells showed a significantly greater proliferation index compared to that of SK-BR-3. The enhanced proliferation of BT-483 cells was abrogated by the knockdown of CTMP [144], indicating that high levels of CTMP expression may be a marker for trastuzumab resistance. The treatment of CTMP-overexpressing BT-483 cells with either Akt inhibitor IV or rapamycin (mTOR inhibitor) resensitized the cells to trastuzumab, while PD98059 (MEK/ERK inhibitor) treatment maintained trastuzumab resistance [144]. These results are consistent with a model in which CTMP overexpression specifically causes trastuzumab resistance by increasing Akt activation, and subsequently mTOR activity [144,147]. CTMP has thus emerged as a potential biomarker for trastuzumab resistance in HER2+ BC.

#### 3.2.2. The NF-κB Pathway

Mucins are heavily O-glycosylated adhesive and/or signaling proteins that function to lubricate and protect luminal tissues either as secreted oligomers or membrane-bound structures [149]. In BC, MUC1-C is a direct activator of the NF-κB p65 transcription factor, a critical component of the NF-κB pathway [150]. Among BCs, MUC-1 is most common in HER2+ cases: it is expressed in 87.5% of HER2+ BC cases, compared to 60.7%, 68.8%, and 56.6% of LumA, LumB, and TNBC cases, respectively [151]. MUC-1 is also highly upregulated in trastuzumab-resistant BC [152] and is generally associated with a poor prognosis [153]. The administration of GO-203, a MUC-1 inhibitor, in trastuzumab-resistant HER2+ cell lines led to a decreased phosphorylation of both HER2 and Akt and the rescue of trastuzumab sensitivity [154]. A more recent study showed that the inhibition of MUC-1 via the WEE1 inhibitor AZD1775 can successfully suppress trastuzumab-resistant HER2+ BC with cancer stem cells (CD44high/CD24low) [155].

Similar to MUC-1, the high expression of MUC-4 in BC is associated with tumor aggressiveness and motility, and HER2+ BC trastuzumab resistance [156,157]. The stimulation of TNFα-expressing HER2+ BC cells results in downstream MUC-4 production, eliciting trastuzumab and T-DM1 resistance. Therefore, MUC4 expression may serve as a predictive biomarker for assessing trastuzumab efficacy and as a tool for guiding combination therapy with TNFα-blocking antibodies alongside anti-HER2 antibodies [158].

#### 3.2.3. Other Pathways

In addition to the PI3K/Akt/mTOR, MAPK, and NF-κB pathways, other oncogenic signaling pathways like Hippo, Notch1, and Wnt/β-catenin also play roles in the HER2-targeted therapy resistance of BC [159].

Recent attention has been redirected to studying breast cancer stem cells (BCSCs) and their effects on HER2+ BC drug resistance. Fueled by signaling pathways such as Wnt/β-catenin, Notch1, and Hedgehog, BCSCs proliferate and differentiate into tumor-stimulating cells, contributing to tumor-stimulating microenvironments via the actions of angiogenesis, extracellular matrix (ECM) formation, and drug discharge pumps. Angiogenesis provides tumors with increased oxygenation for metabolic demands, ECM formation creates a physical barrier that obstructs anti-tumor drugs, and drug discharge pumps reduce the bioavailability of drugs, thereby reducing therapeutic effects [14]. 

Amongst BCSCs, Wnt3 signaling upregulation has been shown to directly contribute to trastuzumab resistance in HER2+ BC. Notch signaling inhibits PTEN, which reduces Akt activity and promotes trastuzumab resistance. Within the Hedgehog pathway, the reduced activity of smoothened (SMO) and glioma-associated oncogene transcription factors (GLI) resulted in reduced BCSC and HER2+ BC invasion, implicating the role of the Hedgehog pathway in stem cell modulation [14].

The combination therapy of anti-HER2 treatment with pathway inhibitors to prevent BCSC function may aid in overcoming drug resistance.

### 3.3. Alterations of the Tumor Microenvironment (TME) as Biomarkers

The tumor microenvironment (TME) comprises the complex ecosystem surrounding the tumor and primarily consists of immune cells, stromal cells, blood vessels, and the extracellular matrix [160]. Recent studies have demonstrated that components of the TME may predict trastuzumab resistance in BC. Specifically, tumor-associated fibroblasts (TAFs), a type of stromal cell in the TME, have been implicated in promoting trastuzumab resistance via fibroblast growth factor receptor 2 (FGFR2) activation in HER2+ BC cells (Figure 2D). In vivo studies demonstrated that the FGFR2 inhibitors ponatinib and infigratinib inhibited tumor growth and resensitized cells resistant to HER2-targeted therapies [161]. TME-mediated mechanisms of resistance to HER2 inhibitors may vary among HER2+ BC subtypes [162]. The activation of the mesenchymal–epithelial transition (MET) factor receptor tyrosine kinase signaling pathway may be more related to resistance to HER2-targeted tyrosine kinase inhibitors (TKIs) in basal-like HER2+ BC cells, while HER2-HER3 heterodimerization is more linked to drug resistance in luminal-like HER2+ BC cells [162]. Additionally, the presence of α-SMA in the TME was identified as a novel biomarker of resistance to trastuzumab in patients with early-stage HER2+ BC [163].

The interaction between tumor cells and the TME is strongly influenced by the PD-1/PD-L1 axis, which is the most extensively studied immune checkpoint blockade (ICB) in HER2-positive BC [164]. PD-L1, expressed in 15% of HER2+ BC patients, is significantly associated with response to the combination therapy of neoadjuvant chemotherapy plus trastuzumab [165]. The treatment of syngeneic mouse tumors transduced to overexpress HER2 with trastuzumab led to the upregulation of PD-L1. HER2-overexpressing human BC cell lines also upregulated PD-L1 in response to trastuzumab treatment, yet only if co-cultured with human peripheral blood mononuclear cells, a response that was inhibited by an IFNγ-neutralizing antibody [166]. These results suggest that trastuzumab-induced PD-L1 upregulation through immune effector cell recruitment may contribute to trastuzumab resistance. These studies have paved the way for the development of combination therapy with HER2 inhibitors and PD-1/PD-L1 inhibitors for the treatment of patients with HER2+ BC.

### 3.4. Other Biomarkers

Additional biomarkers that may be useful for predicting trastuzumab resistance include microRNAs (miRs), which reduce the expression of target genes at the post-transcriptional level, and markers associated with metabolic reprogramming [104,167]. For example, in HER2+ BC cells, FOXO3a regulates miR-128-3p and miR-30a-5p to control IGF2 (Insulin-like Growth Factor 2) and IRS1 expression. The disruption of the FOXO3a-miRNA negative feedback loop leads to the upregulation of IGF2/IGF-1R/IRS1 signaling, resulting in the promotion of trastuzumab resistance [168]. miR-221 is an additional microRNA molecule that promotes trastuzumab resistance and metastasis in HER2+ BC, evincing its potential as a predictive biomarker for HER2-targeted therapy resistance and as a novel target for anti-HER2 combination therapy [169]. The abnormalities in glucose, amino acid, and fatty acid metabolism are also closely linked to HER2 inhibitor therapy in HER2+ BC, and the dysregulated enzymes in these metabolic pathways could be potential markers for the prediction of drug resistance [104].

## 4. Strategies to Overcome Drug Resistance

**Figure 3:**
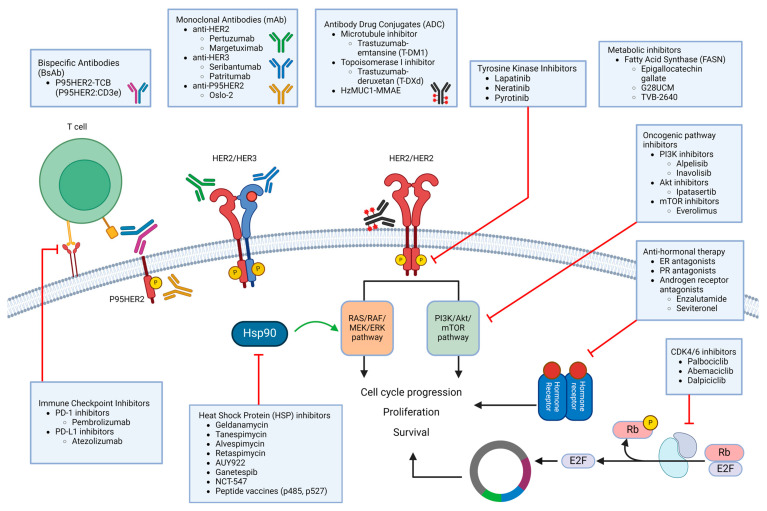
Summary of potential treatment options for HER2+ treatment-resistant breast cancer. Immunotherapies such as new monoclonal antibodies (mAbs), bispecific antibodies, immune checkpoint inhibitors, and antibody–drug conjugates (ADCs) work at the receptor level. Tyrosine kinase inhibitors (TKIs), heat shock protein (HSP) inhibitors, and oncogenic pathway inhibitors target the signaling pathways post receptor. Antihormonal therapies target hormone receptors that interact directly with HER2 signaling, while CDK4/6 inhibitors prevent abnormal cell cycle progression by inhibiting the dissociation of E2F from Rb. Metabolic inhibitors prevent tumors from utilizing fatty acid synthase to enhance proliferative activities. Combination therapies have been explored using oncogenic pathway inhibitors, HSP inhibitors, immune checkpoint inhibitors, metabolic inhibitors, CDK4/6 inhibitors, and anti-hormonal inhibitors, often paired with trastuzumab to enhance anti-tumor activities in trastuzumab-resistant tumors or with other chemotherapies.

The identification of the mechanisms underlying HER2 inhibitor therapy and the discovery of biomarkers predicting HER2 therapy efficacy have paved the way for the development of strategies aimed at overcoming drug resistance (Figure 3, Table 2). Unfortunately, cancer is defined by rapid evolution, and breast cancer embraces evolution just as readily as we evolve therapeutic options in this fight against it.

### 4.1. Tyrosine Kinase Inhibitors (TKIs)

As mentioned before, lapatinib, in combination with capecitabine, is a noncovalently bound, reversible TKI approved for the first-line treatment of HER2+ BC. Acting on EGFR and HER2 tyrosine kinase domains, lapatinib decreased the phosphorylation of 95HER2 and inactivated the downstream Akt and MAPK pathways, inhibiting trastuzumab-resistant tumor growth in mice with HER2+ BC [108,170]. Lapatinib has been shown to also increase peripheral blood mononuclear cell (PBMC)-mediated ADCC when administered with trastuzumab. Furthermore, clinical trials with lapatinib have shown increased tumor-infiltrating lymphocytes, signifying a favorable prognosis in trastuzumab-resistant HER2+ BC patients [171].

Stronger than lapatinib, neratinib is an irreversible pan-TKI that targets EGFR, HER2, and HER4. In p95HER2+ mBC patients, the combination of neratinib + capecitabine demonstrated significantly increased PFS when compared to lapatinib + capecitabine [97]. This effect was particularly amplified in patients who possessed higher levels of HER2, whether full or truncated (p95HER2).

Evaluation of the possibility to switch between TKIs upon the failure of a current TKI has been explored. A small-scale, retrospective study showed that HER2+ BC patients who experienced lapatinib failure experienced increased PFS when treated subsequently with pyrotinib. Pyrotinib treatment in lapatinib-naïve patients also yielded greater PFS compared to lapatinib treatment [172]. These results were potentially due to pyrotinib’s broad inhibition of multiple HERs compared to lapatinib’s HER2-specific inhibition.

### 4.2. Monoclonal Antibodies (mAbs)

The activation of oncogenic pathways, such as the PI3K/Akt pathway, is facilitated by HER2 and HER3 dimerization, leading to anti-HER2 therapy resistance [173]. Pertuzumab has been shown to disrupt such HER2-HER3 dimerization [174]. Despite extensive research, antibodies targeting HER3, including seribantumab and patritumab, have not yet been approved for the treatment of HER2+ BC due to limited therapeutic efficacy.

The monoclonal antibody Oslo-2 has been developed and investigated as a potential treatment specifically targeting the common p95HER2 isoform 611-CTF, which is known for its role in the expression of multiple metastasis-promoting genes such as matrix metalloproteinase 1 (MMP1), angiopoietin-like 4 (ANGPTL4), and the MET tyrosine kinase [175,176]. Oslo-2 functions similarly to trastuzumab, yet exhibits high specificity for p95HER2, and selectively targets HER2+ BCs over other HER2+ cancers. Oslo-2 holds promise for both the diagnostic detection of p95HER2 through IHC, and for the treatment of p95HER2+ BC through the creation of antibody–drug conjugates (ADC) and chimeric antigen receptor-T cell (CAR-T cell) therapies [175].

### 4.3. Bispecific Antibodies

Bispecific antibodies (bsAbs) are antibodies capable of simultaneously binding two distinct antigens, or two distinct epitopes on the same antigen [177]. Engineered T-cell bispecific antibodies (TCBs) possess distinct antigen-binding domains targeting a specific tumor antigen and the epsilon subunit of CD3 (CD3e) expressed on T cells [178]. Binding to both antigens activates T cell receptors (TCRs), leading to the T cell-mediated killing of cancer cells. The P95HER2-TCB, designed with a 2:1 valency (95HER2:CD3e), is designed to activate T cells exclusively in the presence of p95HER2+ tumors. TCBs containing P329G and L234A-L235A (LALA) mutations in the Fc domain prevent unwanted inflammatory responses to these therapeutic antibodies by preventing Fcγ receptor-mediated non-specific immune cell activation [179]. Following in vivo injection of p95HER2-TCB into patient-derived xenografts, an increase of at least two-fold in intratumoral CD8+ T cells was observed [179]. A separate study noted that TCB treatment impaired the growth of intracranial p95HER2+ tumors, suggesting that CD3+ T cells can transport p95HER2-TCB across the blood–brain barrier, a challenge faced by trastuzumab in treating BC brain metastasis [180]. TCBs are still in the preclinical development for the treatment of breast cancer.

### 4.4. Antibody–Drug Conjugates (ADC)

Antibody–drug conjugates (ADC) are therapeutics designed to specifically deliver cytotoxic drugs to cancer cells by conjugating the drugs via a chemical linker to a monoclonal antibody specific for a particular type of cancer cell or tumor. HzMUC1-MMAE is a humanized MUC-1 monoclonal antibody conjugated with monomethyl auristatin (MMAE, microtubule polymerization inhibitor) designed to target BC, as MUC-1 is overexpressed in a variety of cancers, and is a marker for anti-HER2 resistance as described previously [181]. HzMUC1-MMAE effectively halted the growth of trastuzumab-resistant HER2+ BC cell lines and xenografts by inducing cell cycle arrest at the G2/M phase. In addition, the combination treatment of HzMUC1-MMAE with trastuzumab yielded even greater inhibitory effects on cancer growth [181].

ADCs address HER2 inhibitor resistance in HER2+ BC by increasing tumor-specific immunity and reducing immunosuppression in the tumor microenvironment [182]. As described earlier, T-DXd is an ADC of trastuzumab conjugated to the topoisomerase inhibitor deruxtecan. In addition to suppressing tumor growth in mouse cancer models, T-Dxd activated dendritic cell maturation through the upregulation of CD86 and major MHC-II expression, along with the increased expression of PD-L1 and MHC class I expression on tumor cells [183]. These results demonstrate an enhanced anti-tumor immunity that could help overcome trastuzumab resistance, especially if T-Dxd was combined with an anti-PD-1 antibody, to account for the elevated PD-L1 expression induced by TDxd. Similarly, T-DM1 rendered HER2+ BC highly susceptible to the CTLA-4/PD-1 blockade [184] and also overcame lapatinib resistance [96,185]. Although studies have shown a rising statistic of resistance to T-DM1, particularly through continued trastuzumab resistance and altered drug payload internalization and intracellular release [185], ADCs remain a strong and viable treatment for drug-resistant HER2+ BC due to their immunomodulatory effects. There is an increasing interest in evaluating the treatment efficacy of combination therapy using ADCs alongside immune checkpoint inhibitors (ICIs).

### 4.5. Combination Therapy

#### 4.5.1. Combination with Oncogenic Pathway Inhibitors

As discussed earlier, the mutational activation of oncogenic pathways, like the PI3K/Akt/mTOR and MAPK pathways, is strongly associated with resistance to HER2 inhibitors. Targeting these pathways presents a potential strategy for overcoming HER2-targeted therapy resistance in BC. Accumulating preclinical evidence supports the effectiveness of combining PI3K inhibitors with HER2 blockage in the treatment of PIK3CA-mutant HER2+ BC. In MDA-AB-361, an ER+/HER2+ BC cell line bearing PIK3CA-mutations that confer resistance to HER2 therapy, triple-combination therapy with fulvestrant (a selective estrogen receptor degrader), lapatinib, and ipatasertib (an Akt inhibitor) demonstrated enhanced antiproliferative ability in comparison to double-combination therapy with fulvestrant and lapatinib. Similarly, in the ER-/HER2+ BC cell lines HCC1954 and UACC893, both of which contain PIK3CA mutations, combination treatment with PI3K inhibitors alpelisib or GDC-0077 (inavolisib) plus trastuzumab induced anti-proliferative effects and prominent apoptosis compared to single-drug treatments [186]. Additionally, p4EBP1 expression, identified as a potential diagnostic biomarker, may have potential as a prognostic and efficacy-linking biomarker for combination therapy with the PI3K/Akt/mTOR pathway inhibitors [187].

These promising preclinical results prompted clinical trials with the combination therapy of PI3K/Akt/mTOR inhibitors plus HER2 inhibitors for HER2+ BC patients resistant to trastuzumab. In the phase 3 BOLERO-3 trial, a randomized, double-blind, placebo-controlled clinical study, combination therapy with everolimus (an mTOR inhibitor), trastuzumab, and vinorelbine (a chemotherapy drug) significantly improved PFS in patients with trastuzumab-resistant and taxane-pretreated HER2+ mBC [188]. Ongoing clinical trials are evaluating selective PI3K/Akt/mTOR inhibitors in combination with HER2 inhibitors, including alpelisib combined with trastuzumab for the treatment of patients with HER2+ BC bearing PIK3CA mutations, previously treated with HER2 inhibitors (ALPHABET, NCT05063786), and inavolisib in combination with trastuzumab–pertuzumab as maintenance therapy after a chemotherapy regime with docetaxel plus trastuzumab and pertuzumab in patients with HER2+mBC harboring PI3KCA mutations (INAVO122 trial, NCT05894239) [9].

#### 4.5.2. Combination with Heat Shock Protein (HSP) Inhibitors

The HSP90 chaperone complex functionally assists in signal protein folding and protein degradation [189]. It is associated with the turnover and function of several oncogenic client proteins such as BCR-ABL, CDK4, MET, and mutant p53. The “stress hypothesis” suggests that HSP90 is overactive in tumors that depend on its chaperoning functions under hypoxic, nutrient-poor, and highly metabolic environments [190,191]. HSP90 has been implicated as a mediator in the development of tumor drug resistance [192]. Notably, the structural integrity and kinase activity modulation of HER2 and p95HER2 are highly sensitive to HSP90 [193]. These factors make HSP90 an attractive therapeutic target in HER2+ BC treatments.

In vitro and in vivo studies on HSP90 inhibitors have yielded promising results. However, clinical studies investigating the efficacy of the combination therapy of trastuzumab plus HSP90 inhibitors, including geldanamycin, tanespimycin, alvespimycin, retaspimycin, and AUY922, have demonstrated at most a modest effect [194]. A phase I clinical study assessed the efficacy of a combination therapy of ganetespib, a HSP90 inhibitor, plus paclitaxel and trastuzumab in patients with trastuzumab-refractory HER2+ mBC. The overall response rate was 22% (2/9 patients had a partial response), stable disease was seen in 56% of patients (5/9), and the median PFS was 20 weeks (range 8–55) [195]. In addition to showing moderate efficacy in trastuzumab-resistant HER2+ BC, ganetespib was able to reverse secondary lapatinib resistance in HER2+ BC cell lines when combined with lapatinib by reducing STAT3-mediated signaling [196].

A novel C-terminal inhibitor of heat shock protein 90 (HSP90), NCT-547, has shown promising results in trastuzumab-resistant tumor destruction. NCT-547 disrupted downstream survival and proliferation signaling pathways involving Akt and STAT3. NCT-547 additionally decreased EGFR, HER2, HER3, and p95HER2 expression without causing the heat shock factor 1 (HSF1)-induced heat shock response (HSR), which is unfortunately prevalent in N-terminal HSP90 inhibitors [197].

Recently, a HSP90 peptide vaccine has been explored as a therapeutic alternative to direct HSP90 inhibitors in targeting HER2+ BC. Two peptides, p485 and p527, were selected from the most common MHC class II HSP90 epitopes and co-administered with an interferon gene agonist (STING) and an anti-CTLA-4 antibody to induce Th1-mediated immunity. The HSP90 peptides were able to stimulate an anti-HSP90 response in HER2+ tumors in vivo with an increased CD8+ T cell: Treg ratio and decreased HER2+ tumor volumes [198], demonstrating another promising strategy to treat trastuzumab-resistant HER2+ BC.

#### 4.5.3. Combination with Immune Checkpoint Inhibitors (PD-1/PD-L1 Inhibitors)

As mentioned briefly in the TME section, crosstalk exists between HER2 and PD-1/PD-L1 axis in BC [199]. PD-1/PD-L1 expression is most commonly observed in triple-negative breast cancer (TNBC), followed by HER2+ BC, where PD-L1 expression of up to 58%, 53.8%, and 32% has been detected in tumor cells, immune cells, and both cells, respectively [200,201,202]. PD-1/PD-L1 expression correlates with poor clinical outcomes in HER2+ BC [203]. Trastuzumab-mediated upregulation of PD-1 has been suggested as a potential mechanism of trastuzumab resistance [166]. In a preclinical study, anti-PD-1 and anti-CD137 (CD137 is an additional checkpoint inhibitor, also known as 4-1BB) mAbs significantly improved the therapeutic efficacy of anti-HER2 mAb in immunocompetent mice [204]. In a phase 1b-2 clinical trial, the combination therapy of PD-1 inhibitor pembrolizumab plus trastuzumab showed some degree of clinical benefit, with an overall response rate (ORR) of about 15% in trastuzumab-resistant HER2+ mBC bearing PD-L1 expression [205]. However, in the phase 2 randomized KATE2 trial, the combination therapy of atezolizumab (a PD-L1 inhibitor) and trastuzumab emtansine (T-DM1) in PD-L1 positive previously treated HER2+ mBC did not demonstrate a significantly improved PFS when compared to trastuzumab plus placebo [206]. Because immune checkpoint inhibitors (ICIs) are primarily effective in early-stage tumors [207], the ASTEFANIA clinical trial (NCT04873362) is investigating the combination therapy of PD-1/PD-L1 inhibitor (atezolizuman) plus T-DM1 over T-DM1 plus placebo in early-stage HER2+ BC and residual disease after neoadjuvant HER2-targeted therapy plus chemotherapy. The trial’s follow up is planned for approximately 10 years from the last patient enrolled [208]. Although ICIs are generally safe and well tolerated, their clinical efficacy in HER2+ BC, particularly in cases without PD-L1 expression, remains uncertain. Further clinical trials are needed to clarify the clinical benefits of combination therapy with ICIs and HER2 inhibitors in the treatment of HER2+ BC.

#### 4.5.4. Combination with Metabolic Inhibitors

Metabolic reprogramming, a hallmark of cancer, contributes significantly to HER2 inhibitor resistance. One aspect of this reprogramming involves the upregulation of glycolysis and fatty acid metabolism, which has been linked to trastuzumab resistance [209]. Fatty acid synthase (FASN), the key enzyme involved in fatty acids (FAs) biosynthesis, is frequently upregulated in HER2+ BC. FASN overexpression leads to the increased uptake of FAs, tumor cell proliferation, survival, HER2 activation, and resistance to anti-HER2 therapy [210,211]. Similarly, the elevated expression of CD36, a FA transporter, is associated with poor prognosis and resistance to anti-HER2 therapy in BC [212]. In the NeoALTTO phase 3 trial, involving 180 patients with HER2+ BC, intratumor CD36 gene expression was identified as a predictor of worse clinical outcomes in early-stage HER2+ BC treated with trastuzumab-based neoadjuvant therapy [213].

The observation that the FASN blockade leads to apoptosis in HER2+ BC cells has led to preclinical investigations using the dual FASN and HER2 signaling blockade in BC models resistant to anti-HER2 drugs [214]. Epigallocatechin gallate (EGCG), a FASN inhibitor extracted from green tea [215], reduced cell proliferation, ATP production, and AKT activity in trastuzumab-resistant HER2+ BC cells. Moreover, EGCG in combination with trastuzumab provided a novel strategy for treatment, particularly because EGCG can cross the blood–brain barrier [216]. Additionally, the anti-FASN compounds EGCG and G28UCM plus pertuzumab showed synergistic interactions in HER2+ BC cells resistant to lapatinib, trastuzumab, and lapatinib. This combination also improved treatment outcomes in orthoxenopatient mice receiving tumors derived from a BC patient who relapsed on trastuzumab- and lapatinib-based therapy [217]. Finally, the FASN inhibitor TVB-2640 is currently being evaluated in a phase II clinical trial in combination with paclitaxel and trastuzumab or endocrine therapy for the treatment of patients with HER2+ mBC (NCT03179904). Thus, targeting metabolic reprogramming, particularly fatty acid synthase (FASN) inhibition, in combination with standard HER2-targeted therapies represents a promising approach for overcoming resistance to anti-HER2 therapy in HER2+ breast cancer.

#### 4.5.5. Combination with CDK4/6 Inhibitors

A growing body of evidence suggests that the cyclin D1/CDK4/6/pRb pathway plays a role in resistance to HER2-directed treatments [218,219]. The inhibition of CDK4/6 has been shown to suppress the phosphorylation-dependent inhibition of the tumor suppressors Rb and TSC2, thereby reducing mTORC1 activity. Moreover, CDK4/6 inhibitors sensitized patient-derived xenograft tumors to HER2-targeted therapies and delayed tumor recurrence in a transgenic model of HER2+ BC [219]. Building on this promising data, clinical trials are investigating combination therapy with CDK4/6 inhibitors and HER2 inhibitors in HER2+ BC patients. The results from the phase II SOLTI-1303 PATRICIA trial demonstrated that CDK4/6 inhibitor palbociclib in combination with trastuzumab was safe and exhibited promising survival outcomes in ER+/HER2+ mBC previously treated with trastuzumab [220]. In the randomized phase II monarcHER trial, the combination therapy of CDK4/6 inhibitor abemaciclib plus trastuzumab, with or without fulvestrant, significantly improved PFS in women with hormone receptor-positive, HER2-positive, advanced BC compared to standard-of-care chemotherapy plus trastuzumab, and this combination regimen was safe and tolerable [221]. Most recently, in a single-arm phase II trial, the treatment of HER2+ mBC patients with the CDK4/6 inhibitor dalpiciclib and HER2 TKI pyrotinib showed promising activity and manageable toxicity [222]. These results underscore the potential clinical benefit of combination therapy with CDK4/6 and HER2 inhibitors in combating anti-HER2 drug resistance in HER2+ BC.

#### 4.5.6. Combination with Anti-Hormonal Therapy

The crosstalks between HER2 and hormonal signaling pathways, triggered by the receptors for estrogen (ER), androgen (AR), and progesterone (PGR), are implicated in resistance to both anti-hormone and anti-HER2 therapies [223,224]. Therefore, combination therapies consisting of anti-HER2 inhibitors plus anti-hormone drugs may improve outcomes for patients with HR+/HER2+ BC. Both in vitro and in vivo models have demonstrated that combination therapies with both HER2-directed and endocrine therapies lead to more significant tumor regression compared to anti-HER2 therapy alone [225,226]. Consequently, several clinical trials have explored the efficacy of combining endocrine- and HER2-targeted therapies in various settings, including neoadjuvant, extended adjuvant, and mBC [227]. These trials have demonstrated that combined endocrine and anti-HER2 therapies result in enhanced therapeutic efficacy and safety in patients with HR+/HER2+ or ERBB2-mutated BCs [227]. For example, the phase 2 PAMELA clinical trial showed that dual anti-HER2 and endocrine therapies increased the partially conditional rate-based (pCRB) and pCR, especially in HER2+/HR+ BC [228]. Furthermore, given the positive correlation between ER and HER2 expression in HER2 non-overexpressing BCs [229], the potential clinical benefit of combination therapy using anti-HER2 drugs and anti-hormone drugs warrants investigation in BC patients lacking HER2 overexpression in future studies.

Androgen has emerged as a potential biomarker for predicting a pathological complete response in HER2+ BC treated with trastuzumab and pertuzumab [230]. AR antagonists, such as enzalutamide or seviteronel, exhibited a synergistic effect when co-administered with anti-HER2 drugs, like everolimus and trastuzumab, in BC cells resistant to anti-HER2 therapy [231]. A clinical trial has demonstrated the utility of enzalutamide plus trastuzumab in patients with HER2+/AR+ mBC, yet a remaining challenge is to identify biomarkers that can stratify which patients could benefit from the treatment [232].

Table 3 shows selective clinical trials with combination therapies for the treatment of HER2+ breast cancer.

## 5. Conclusions and Future Perspectives

The advent of HER2-targeted therapies, spearheaded by trastuzumab’s approval two decades ago, marked a major breakthrough for the treatment of patients with aggressive HER2+ breast cancer (BC) [8]. Subsequent FDA approvals of monoclonal antibodies (mAbs), tyrosine kinase inhibitors (TKIs), antibody–drug conjugates (ADCs), and immunotherapy have further expanded the therapeutic arsenal against HER2+ BC. Despite these achievements, resistance to anti-HER2 therapy persists via diverse mechanisms. Combination therapy enhances the treatment efficacy and may overcome drug resistance to anti-HER2 therapy in BC; however, it has potential drawbacks. By virtue of its nature, combination therapy increases the risk of side effects, and interaction between drugs may lead to variability in drug exposure. Such therapy is often also not cost effective. Additionally, barriers exist regarding the application of trastuzumab-based anti-HER2 therapy, due to the limitations of insurance coverage, drug availability and cost, and geographical and socioeconomic disparities among breast cancer patients. This indicates that the introduction of a biosimilar to trastuzumab may alleviate barriers to treatment and increase patient access to HER2-targeted therapy [233]. The identification of biomarkers that might predict HER2-inhibitor resistance and the development of new platforms and more effective therapies is still ongoing. To address these challenges, investigators are exploring dual-targeted or even multi-targeted therapy in clinical trials, aiming to improve clinical outcomes and minimize side effects. The evolving landscape of anti-HER2-based therapy will continue to expand into novel therapeutic avenues, offering renewed hope for patients battling HER2+ BC.

## Figures and Tables

**Table 2:** Mechanisms of action and resistance for FDA-approved HER2 inhibitors.

Anti-HER2 Regimen	Mechanism of Action	Mechanism For/Against Resistance
monoclonal antibodies (mAbs)	binds to ECD of HER2 receptor, inhibiting downstream signaling pathways (PI3K, MAPK) and inducing immune responses (ADCC)	challenged by various mechanisms of resistance, including receptor structural mutations and mutations in downstream signaling pathways
trastuzumab	binds to ECD IV more strongly inhibits downstream signaling pathways (PI3K/AKT) inhibits ligand-independent HER2/HER3 dimerization; inhibits HER2/HER2 homodimerization	structural mutations prevent binding; mutations in downstream signaling pathways bypass trastuzumab binding altogether
pertuzumab	binds to ECD II inhibits ligand-dependent HER2 dimerization	designed to delay trastuzumab resistance, potentially by enhancing trastuzumab-induced ADCC; however, similarly susceptible to resistance
margetuximab	binds to ECD IV structurally similar to trastuzumab; Fc domain of antibody optimized to enhance ADCC	designed to delay trastuzumab resistance by enhancing ADCC, but has not proven very effective
antibody–drug conjugates (ADCs)	deliver potent cytotoxic drugs specifically to antibody targets (HER2+ cells)	can overcome trastuzumab resistance via increased anti-tumor immunity
T-DM1	includes the anti-tumor activities of trastuzumab and DM1, which inhibits microtubule polymerization once the HER2-T-DM1 complex is internalized via receptor-mediated endocytosis and lysed	resistance can arise from mechanisms of resistance to trastuzumab, dysfunctional intracellular trafficking (which reverses internalization of HER2-T-DM1 complexes), and impairment of DM1-mediated toxicity
T-DXd	includes anti-tumor activities of trastuzumab and DXd, a topoisomerase inhibitor in contrast to T-DM1, antibody–drug linker is cleavable, allowing DXd action in nearby non-HER2 expressing cells in contrast to T-DM1, higher drug-to-antibody ratio	decrease in HER2 expression and payload resistance confers resistance to T-DXd
tyrosine kinase inhibitors (TKIs)	block intracellular TK domain of HER receptors, preventing phosphorylation and inhibiting downstream signaling pathways; can cross BBB	mutations to TK domain of HER2 receptor may confer resistance; however, are effective against many mechanisms of resistance to mAbs and ADCs
lapatinib	reversibly binds to TK domain of HER1/HER2 receptors	
neratinib	irreversibly binds to TK domain of HER1/HER2/HER4 receptors	
tucatinib	reversible; binds specifically to HER2 receptor	

ECD: extracellular domain; ADCC: antibody-dependent cellular cytotoxicity; TK: tyrosine kinase.

**Table 3:** Selective clinical trials with combination therapies for treatment of HER2+ BC.

Anti-HER2 Regimen	In Combination With	Clinical Trial	Treatment Setting
	P13K/Akt/mTOR inhibitors		
trastuzumab (mAb)	everolimus (mTOR inhibitor) + vinorelbine (chemo): improved PFS	NCT01007942	trastuzumab-resistant and taxane-pretreated, HER2+ mBC
trastuzumab (mAb)	alpelisib (PI3K inhibitor)	NCT05063786 *	HER2+ BC bearing PIK3CA mutations, previously treated with HER2 inhibitors
	HSP inhibitors		
trasuzumab (mAb)	ganetespib + paclitaxel (chemo): clinical benefit rate of 44%	NCT02060253	Trastuzumab-refractory HER2+ mBC
	PD-1/PD-L1 inhibitors		
trasuzumab (mAb)	pembrolizumab: some degree of clinical benefit; ORR of 15%	NCT02129556	Trastuzumab-resistant mBC, PD-L1+
T-DM1 (ADC)	atezolizumab: no obvious improvement of PFS	NCT02924883	HER2+ mBC, PD-L1+, previously treated
	metabolic inhibitors		
trasuzumab (mAb)	TVB-2640 (FASN inhibitor) + paclitaxel or endocrine therapy	NCT03179904 *	HER2+ mBC
	CDK4/6 inhibitors		
trasuzumab (mAb)	palbociclib: promising survival outcomes	NCT02448420	ER+/HER2+ mBC, resistant to HER2 inhibitor
trasuzumab (mAb)	abemaciclib: improvement of PFS	NCT02675231	HR+/HER2+ advanced BC
pryotinib (TKI)	dalpiciclib: promising activity, manageable toxicity	NCT05328440	HER2+ advanced BC
	hormonal therapy		
lapatinib (TKI) + trasuzumab (mAb)	letrozole or tamoxifen: increased pCR + pCRB rates in HER2+/HR+ group	NCT01973660	stage I-IIIA HER2+ BC
trasuzumab (mAb)	enzalutamide (androgen receptor inhibitor): CBR24 of 24%, median PFS of 3.4 months	NCT02091960	HER2+ BC, previously received ≥ 1 anti-HER2 regimen

mBC: metastatic breast cancer; mAb: monoclonal antibody; ADC: antibody–drug conjugate; TKI: tyrosine kinase inhibitor; HSP: heat shock protein. ORR: overall response rate; PFS: progression-free survival; pCR: pathological complete response; pCRB: pathological complete response in the breast; * ongoing.
